# CircC3P1 attenuated pro‐inflammatory cytokine production and cell apoptosis in acute lung injury induced by sepsis through modulating miR‐21

**DOI:** 10.1111/jcmm.15685

**Published:** 2020-08-26

**Authors:** Wen‐Yang Jiang, Jie Ren, Xing‐Hua Zhang, Zi‐Long Lu, Hao‐Jie Feng, Xiao‐Li Yao, Dong‐Hang Li, Rui Xiong, Tao Fan, Qing Geng

**Affiliations:** ^1^ Department of Thoracic Surgery Renmin Hospital of Wuhan University Wuhan China; ^2^ Department of Otorhinolaryngology Head and Neck Surgery Renmin Hospital of Wuhan University Wuhan China; ^3^ Department of Breast and Thyroid Surgery Renmin Hospital of Wuhan University Wuhan China

**Keywords:** acute lung injury, circC3P1, sepsis

## Abstract

Acute lung injury (ALI) induced by sepsis is characterized by an inflammatory process related to the up‐regulation of inflammatory cytokines and chemokines. In the present study, we explored the role of circC3P1 in sepsis‐induced ALI in vitro and in vivo. The caecal ligation and puncture (CLP)‐induced sepsis model was established through CLP surgery. Forty adult male C57BL/6 mice were randomly assigned into sham, CLP, CLP + vector and CLP + circC3P1 (each n = 10). Primary murine pulmonary microvascular endothelial cells (MPVECs) were transfected with circC3P1 or empty vector 24 hours prior to LPS treatment via Lipofectamine 2000. The expressions of circC3P1, tumour necrosis factor‐α (TNF‐α), interleukin‐6 (IL‐6) and IL‐1β were evaluated after 6‐h LPS treatment. Cell apoptosis was evaluated via flow cytometry. The CLP group demonstrated pulmonary morphological abnormalities, increased concentrations of TNF‐α, IL‐6 and IL‐1β in the lung tissue, compared with the sham group. MPVECs treated with LPS significantly elevated TNF‐α, IL‐6 and IL‐1β levels and increased cell apoptosis than that in the control group. The circC3P1 overexpression in sepsis‐induced ALI mice attenuated pulmonary injury, inflammation and apoptosis. Besides, circC3P1 revealed anti‐inflammatory and anti‐apoptotic effect in MPVEC‐treated LPS. CircC3P1 overexpression reduced cell apoptosis and pro‐inflammatory cytokines levels via down‐regulating miR‐21. CircC3P1 attenuated pro‐inflammatory cytokine production and cell apoptosis in ALI induced by sepsis through modulating miR‐21, indicating that circC3P1 is a promising therapeutic biomarker for sepsis‐induced ALI.

## 1 INTRODUCTION

Sepsis is a systemic inflammatory response syndrome, which is triggered by infection with pathogenic bacteria, viruses or fungi.^1^ In severe septic patients, multi‐organ dysfunction is the major cause of death.^2^ Acute lung injury (ALI), one of the most common severe sepsis complications, is a syndrome of severe acute respiratory failure.^3^ Nevertheless, there is no recommended standard treatment for ALI.

ALI induced by sepsis is characterized by an inflammatory process, which is related to the up‐regulation of inflammatory cytokines and chemokines.^4^ Increased interleukin‐1b (IL‐1β) was observed in the plasma and bronchoalveolar lavage fluids in some ALI models.^5^ Another pathophysiology of ALI is the abnormal apoptosis of pulmonary cells.^6^ Hence, blocking apoptotic signalling and inflammatory responses in the lung might act as a promising therapy to improve sepsis‐induced ALI.

Lipopolysaccharide (LPS) is a group of polar lipid heads (lipid A) and chain repeating disaccharides present in the outer membrane of Gram‐negative bacteria.^7^ In serum, LPS binds to α‐specific LPS‐binding protein (LBP) to form a type of LPS,^8^ which activates the LBP complex of CD14/TLR4 receptors on monocytes, macrophages and other cells, triggering the production of inflammatory mediators.^9^ LPS is a vital medium for sepsis, and the systemic administration of the body's reaction to Gram‐negative bacteria leads to bacterial septicaemia.^10^ LPS activates the pathway of polymorphonuclear leucocyte (PMN) migration in the circulation through enhancing the mechanical properties and migration activities of leucocytes.^10^ This allows leucocytes to migrate into the lung tissue, releasing chemotactic cytokines into the bronchoalveolar lavage fluid (BAL), and thus chemotaxis the PMN expressed by factor receptors is guided into the lung tissue.^11^

This process requires the contact of adhesion molecules between PMN and the lung endothelium.^11^ Once PMN adheres to the pulmonary vascular wall, it begins to migrate through the endothelium to the lung interstitium and across the epithelium to the alveolar space.^11^ The two migration steps are regulated by adhesion molecules and chemokine receptors, accompanied by cytoskeletal reorganization of PMN, endothelial cells and epithelial cells.^12^ Research evidence shows that exposing mice to the LPS environment can induce a large amount of PMN migration into the lung, which can cause ALI typical symptoms, including increased microvascular permeability, cytokine release and damaged lung structure.^8^

Circular RNAs (circRNAs), a novel type of noncoding endogenous RNAs, can modulate the gene expression post‐transcriptionally via serving as miRNA sponges.^13^ Recently, Li *et al* have reported the changes of circRNAs level and potential molecular mechanism in the ALI mice model by microarray analysis, suggesting that circRNAs make a vital contribution to the development of ALI.^14^ CircC3P1 was derived from exons 27 to 29 of complement component 3 precursor pseudogene (C3P1).^15^ Zhong *et al* found that circC3P1 was decreased and exerted an inhibitory effect in liver tumour.^15^ Research evidence revealed that circC3P1 suppressed the activity of kidney cancer cell through modulating miR‐21/PTEN axis.^16^ However, the potential role of circC3P1 and miR‐21 in ALI remains unclear.

In the present study, we explored the role of circC3P1 in sepsis‐induced ALI in vivo and in vitro. Furthermore, we investigated the effect of circC3P1 on lung tissue injury, inflammatory cytokines and cell apoptosis. Such findings may shed new lights on the treatment of ALI induced by sepsis.

## 2 METHODS

### 2.1 Establishment of CLP‐induced sepsis mice model

Forty adult male C57BL/6 mice were obtained from Shanghai Sippr‐BK laboratory animal Co. Ltd. All mice were caged under measured environment (23 ± 2°C, 12/12‐hours light‐dark cycle, 55% relative humidity) with water and food ad libitum. All experiments were performed based on the protocols authorized by Renmin Hospital of Wuhan University Animal Care and Use Committee. The caecal ligation and puncture (CLP)‐induced sepsis model was established through CLP surgery as described before.^17^ Briefly, mice were fixed on the operating table after anesthetizing through intraperitoneal injection with 10% chloral hydrate. A midline incision (4 mm) was made to expose the caecum which was sutured with a 3‐0 silk suture to a distance of 10 mm from the tip and punctured using a 20‐gauge needle at a distance of 5 mm from the ligation. Then, the bowel was repositioned carefully, and the peritoneum and skin were sutured using utilizing sterile sutures. All mice were subcutaneously administered 5 mL/100 g saline for fluid resuscitation after surgery. Ten sham‐operated mice received a similar surgery, but they did not undergo ligation or puncture of the caecum.

### 2.2 Plasmid DNA delivery

CLP‐induced sepsis mice were randomly assigned into CLP, CLP + vector and CLP + circC3P1 (each n = 10). The vector (negative control) and circC3P1 reagents were provided by GenePharm (Shanghai, China), using polyethyleneimine nanoparticles (Sigma‐Aldrich) to mix them based on previous methods.^18^ A volume of 200 µL mixture with 5 nmol/L circC3P1 or vector was injected intravenously into the animals’ tail vein 7 days before the CLP surgery. Mice were euthanized 24 hours after operation via carbon dioxide asphyxia. Lungs were collected for further research.

### 2.3 Histological analysis

For histopathological examination, lung tissue samples from the same portion of mice were fixed in 4% paraformaldehyde. Paraffin‐embedded tissues were cut into 5 μm sections, staining with haematoxylin‐eosin (HE). Lung injury scores were evaluated by researchers who were not familiar with experiments as published previously.^19^ Each slide was scored for five randomly selected fields at 400x magnification.

### 2.4 Cell culture and transfection

Primary murine pulmonary microvascular endothelial cells (MPVECs) were fostered in DMEM (Gibco, USA) according to the previous method.^20^ To establish the overexpression circC3P1 vector, the circC3P1 sequence was cloned into PKCDH circle vector (RiboBio, China). The vector (negative control) was provided by GenePharm (Shanghai, China). We transfected circC3P1 and empty vector into cells via Lipofectamine 2000 (Invitrogen, USA). After 24‐hours transfection, MPVECs were exposed to lipopolysaccharide (LPS, 1 mg/mL) purchased from Sigma‐Aldrich. The expressions of circC3P1, TNF‐α, interleukin‐6 (IL‐6) and IL‐1β were evaluated after 6‐h LPS treatment.

### 2.5 Quantitative RT‐PCR

The RNAiso Plus (Takara, China) was utilized to extract total RNA from MPVECs and mice lung tissues. The PrimeScript™ RT Master Mix (Takara, China) was employed to reversely transcribe RNA to cDNA as the manufacturer's instructions. The circC3P1 and p‐AKT/AKT levels were measured via TB Green^®^ Premix Ex Taq™ (Takara, China) according to the protocol. β‐actin was as an internal control. The data were analysed through 2^−ΔΔCt^ approach. Primers were listed in Table S1.

### 2.6 Detection of pro‐inflammatory cytokines

We prepared the total protein extraction from MPVEC lysates and mice lung tissues to measure pro‐inflammatory cytokines levels. ELISA kits (R&D Systems, USA) were applied to evaluate TNF‐α, IL‐6 and IL‐1β concentrations following the manufacturer's protocol.^21^

### 2.7 Flow cytometry

Cell apoptosis was examined using the Annexin V‐FITC Apoptosis Detection Kit (Abcam) based on the manufacturer's instructions. In short, MPVECs were planted in 6‐well plates and grown to 70%‐80% confluency. After above‐mentioned transfection and LPS stimulation for 24 hours, cells were centrifuged and resuspended in buffer solution (500 µL) to the density of 5 × 10^5^ cells/mL. Next, cells were fostered with annexin V‐FITC (5 μL) and propidium iodide (PI, 5 μL) for 15 minutes in the dark. Apoptosis was quantified through BD FACS software.

### 2.8 Western blot

Proteins extracted from MPVEC lysates were analysed by 8% SDS‐PAGE gel and transferred to PVDF membranes as previously described.^22^ Then, membranes were incubated with primary antibodies (1:1000, Abcam) for 6 hours at 4°C, including p‐AKT, AKT and β‐actin. The membrane was rinsed with TBST for 3 times and fostered with goat anti‐rabbit secondary antibody (1:5000, Abcam) at 25°C for an hour. The Bio‐Rad Image LabTM Software was performed on protein quantification.

### 2.9 Statistical analysis

All the current study data, displayed as mean ± standard deviation, were analysed applying SPSS 25.0 through two‐tailed Student's t test or one‐way ANOVA. The results were considered statistically significant when *P < *.05.

## 3 RESULTS

### 3.1 CircC3P1 alleviated mice lung injury induced by sepsis after CLP surgery and suppressed the pro‐inflammatory cytokines expressions in the lung tissues of mice with CLP

**FIGURE 1:**
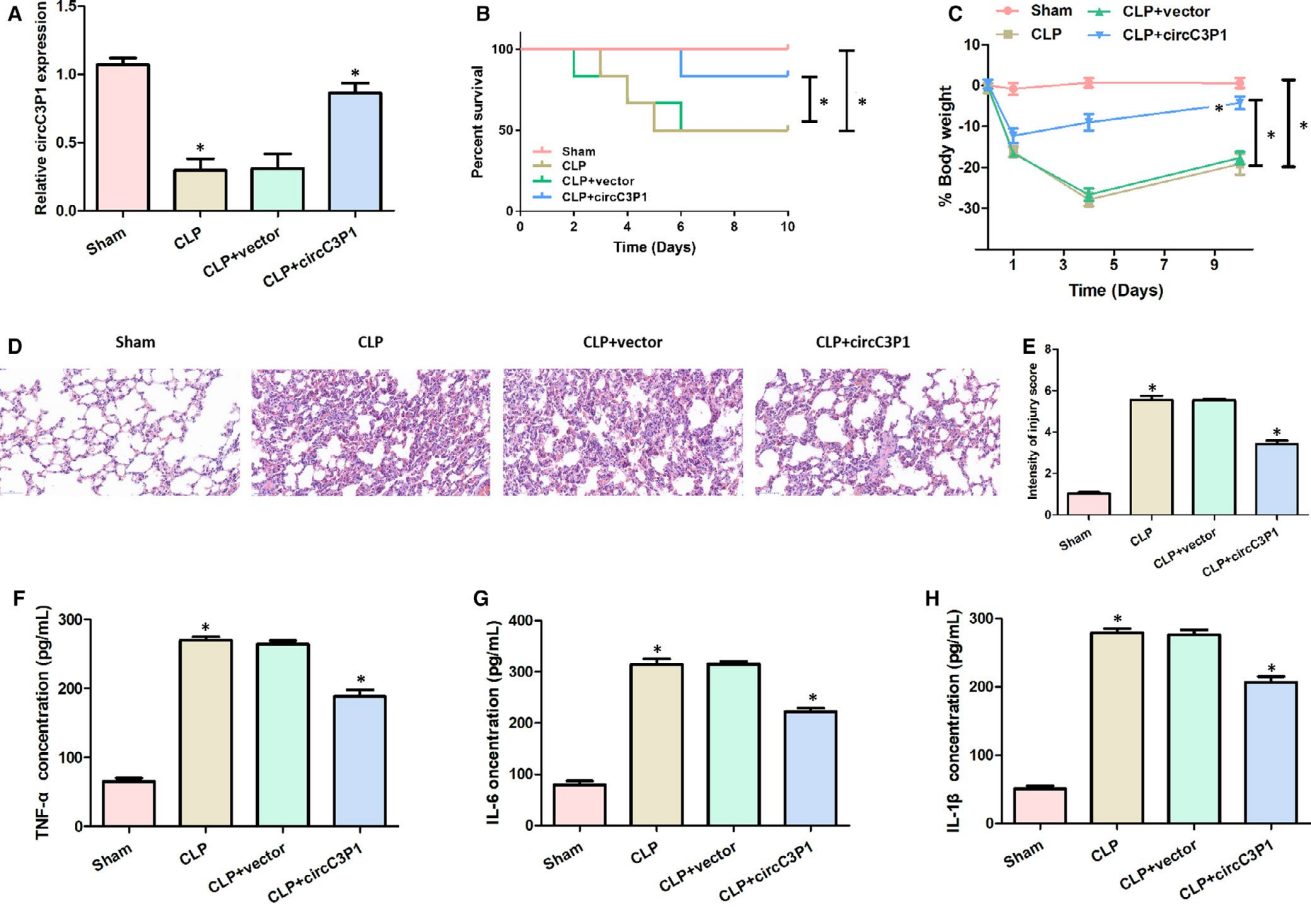
CircC3P1 alleviated mice lung injury induced by sepsis after CLP surgery and suppressed the pro‐inflammatory cytokines expressions in the lung tissues of mice with CLP. A, Relative circC3P1 expression in four groups. B, Per cent survival in four groups. C, Body weight in four groups. D, Representative histological images in four groups. E, Intensity of injury score in four groups. F‐H, The concentration of TNF‐α (F), IL‐6 (G) and IL‐1β (H) in four groups.*P < 0.05

To explore the effect of circC3P1 in ALI induced by sepsis, CLP‐induced sepsis mice model was established. Mice were intravenously injected circC3P1 to induce circC3P1 expression before the CLP treatment. Figure 1A indicated that the CLP group had evidently lower circC3P1 expression in the lung tissues compared with the sham group. The circC3P1 expression in the CLP + circC3P1 group increased remarkably than that in the CLP group and the CLP + vector group. Moreover, the per cent survival and body weight were significantly increased in the CLP + circC3P1 group, compared with the CLP group and the CLP + vector group in Figure 1B,C. As presented in Figure 1D, the sham group presented morphologically normal alveoli. In contrast, the CLP group demonstrated collapsed alveolar sacs, as well as thickened alveolar walls and septa. Besides, vascular congestion and bleeding were seen in the CLP group. No significant difference was observed in the histopathological characteristics between the CLP group and the CLP + vector group. As demonstrated in Figure 1E, the CLP group reported significantly higher intensity of injury score than the sham group, whereas the CLP + circC3P1 group had a remarkably lower injury score as compared to the CLP group. These findings showed that circC3P1 alleviated mice lung injury induced by sepsis after CLP operation.

As presented in Figure 1F‐H, the pro‐inflammatory cytokines expressions, including TNF‐α, IL‐6 and IL‐1β, were remarkably higher in the CLP group than those in the sham group. However, significantly decreased TNF‐α, IL‐6 and IL‐1β levels were observed in the CLP + circC3P1 group compared with the CLP group. These data revealed that circC3P1 suppressed the production of pro‐inflammatory cytokines in the lung tissues of mice with CLP.

### 3.2 CircC3P1 overexpression decreased cell apoptosis in MPVECs induced by LPS

**FIGURE 2:**
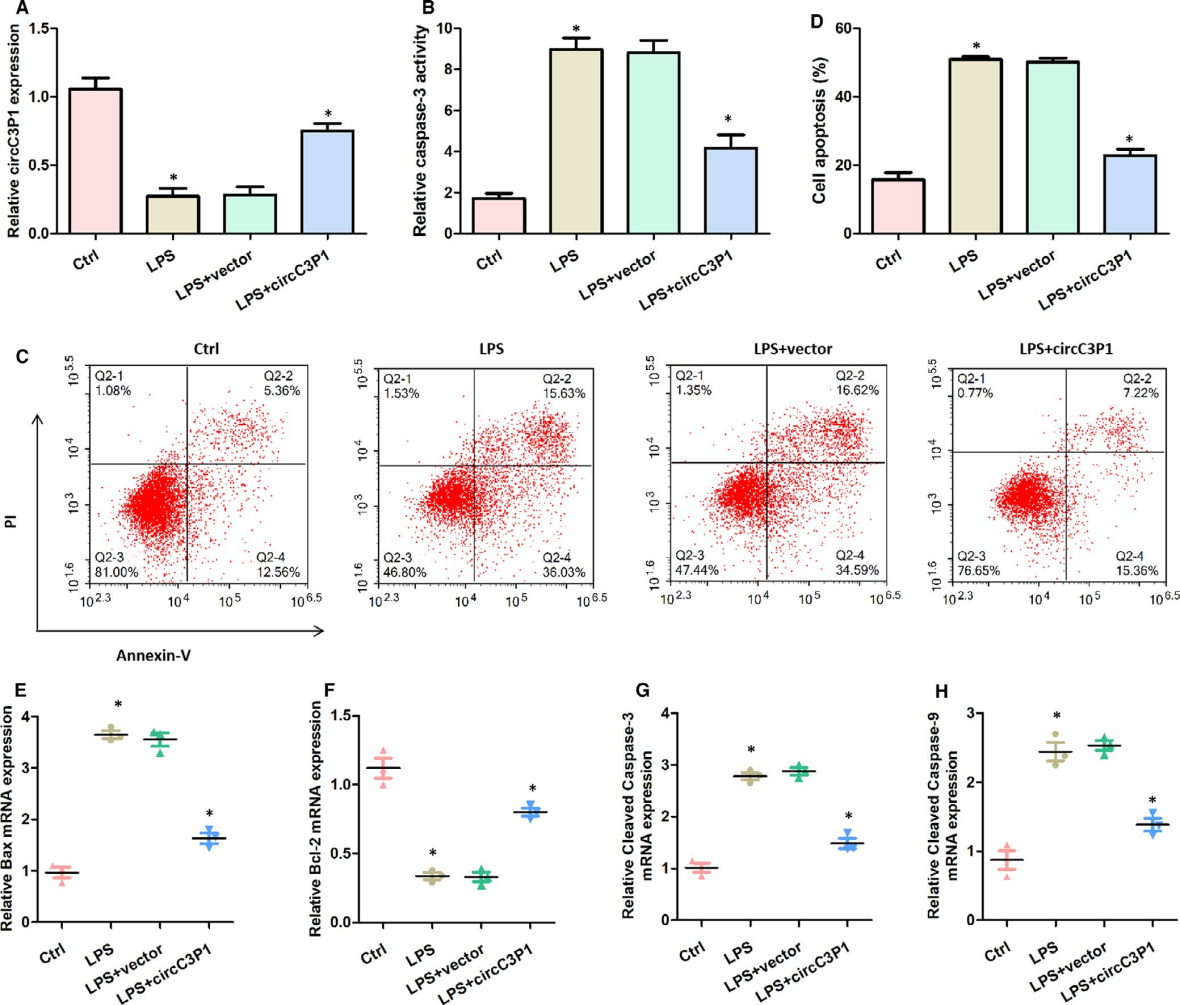
CircC3P1 overexpression decreased cell apoptosis in MPVECs induced by LPS. A, Relative circC3P1 expression in four groups. B, Relative caspase 3 activity in four groups. (C,D) Measurement of the apoptosis rates of MPVECs cells by flow cytometry. E, Relative Bax mRNA expression in four groups. F, Relative Bcl‐2 mRNA expression in four groups. G, Relative cleaved caspase 3 mRNA expression in four groups. H, Relative cleaved caspase 9 mRNA expression in four groups.*P < 0.05

To simulate lung injury, MPVECs were treated with LPS in vitro. After LPS treatment, compared with the control group, the relative circC3P1 expression was lower in the LPS group. Nevertheless, a significant increase level of circC3P1 was witnessed in the LPS + circC3P1 group as compared to the LPS group, as displayed in Figure 2A. The results of flow cytometry indicated that the cell apoptosis in MPVECs was significantly higher in the LPS group, compared with the control group. Nevertheless, the cell apoptosis ratio was efficiently decreased in the LPS + circC3P1 group as compared to the LPS group, as shown in Figure 2C,D. The relative caspase 3 activity, Bax, cleaved caspase 3 and cleaved caspase 9 mRNA expression were remarkably increased and the relative Bcl‐2 mRNA expression was significantly decreased in the LPS group than those in the control group, whereas circC3P1 expression reversed this effect, as shown in Figure 2E‐H. These findings showed that circC3P1 played an anti‐apoptotic role in vitro.

### 3.3 CircC3P1 overexpression restrained p‐AKT/AKT expression and reduced pro‐inflammatory cytokines levels in LPS‐treated MPVECs

**FIGURE 3:**
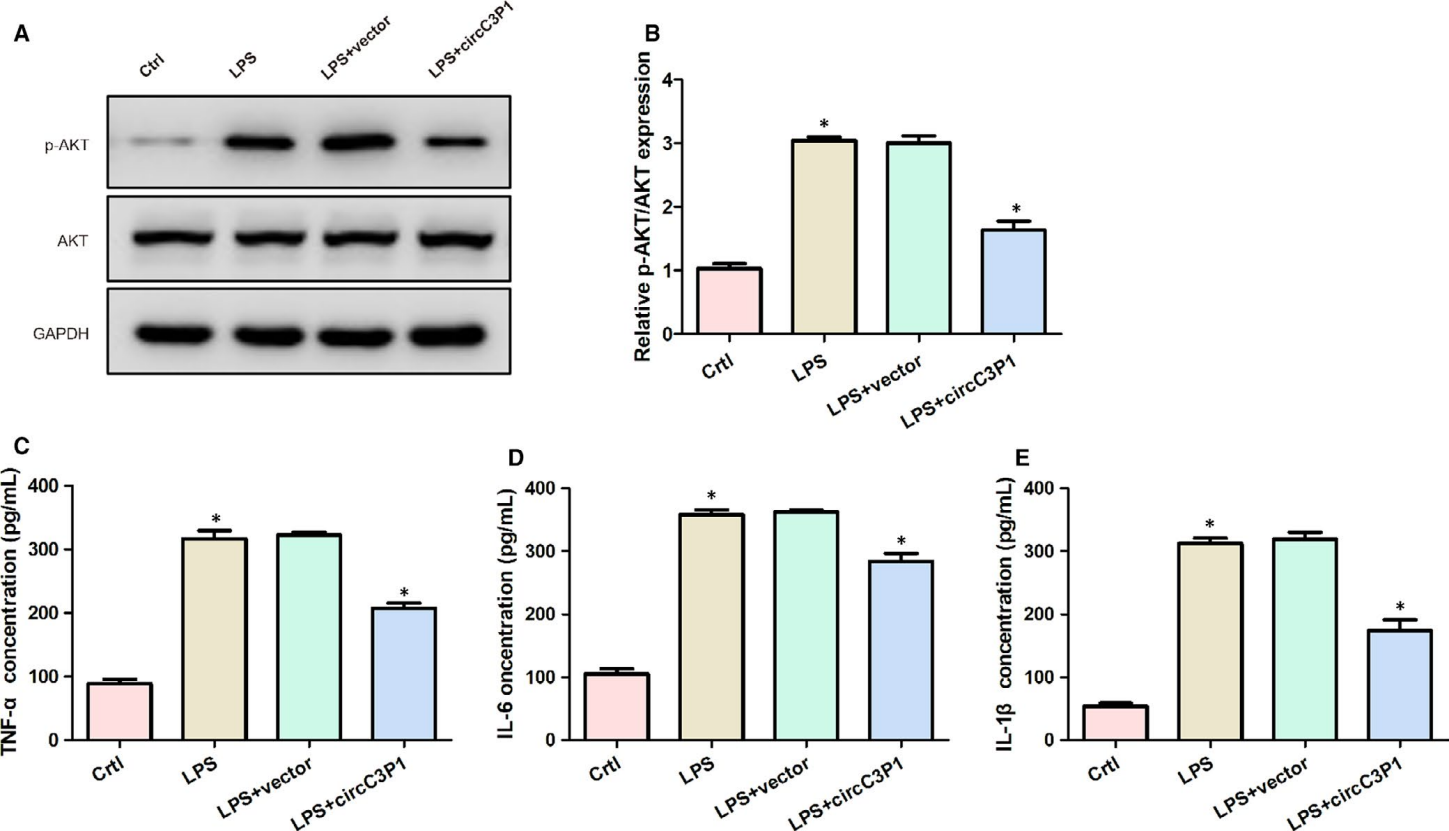
CircC3P1 overexpression restrained p‐AKT/AKT expression and reduced pro‐inflammatory cytokines levels in LPS‐treated MPVECs. A, The expression of p‐AKT and AKT protein in four groups. B, Relative expression of p‐AKT/AKT mRNA in four groups. C‐E, The concentration of TNF‐α (C), IL‐6 (D) and IL‐1β (E) in four groups. *, *P* < 0.05

As can be seen from Figure 3A,B, the LPS‐treated group showed remarkably higher p‐AKT and AKT protein levels, as well as relative p‐AKT/AKT expression, in comparison with the control group. Nevertheless, the circC3P1 overexpression evidently decreased the p‐AKT/AKT mRNA and protein levels as compared to LPS + vector group. Figure 3C‐E showed that the concentrations of TNF‐α, IL‐6 and IL‐1β were significantly promoted through the treatment of LPS in MPVECs. Also, LPS‐treated MPVECs transfected with control vector demonstrated similar TNF‐α, IL‐6 and IL‐1β concentrations. However, the circC3P1 overexpression significantly decreased TNF‐α, IL‐6 and IL‐1β levels in MPVECs treated with LPS. In summary, these data suggest that circC3P1 overexpression suppressed p‐AKT/AKT and reduced pro‐inflammatory cytokines levels in LPS‐treated MPVECs.

### 3.4 CircC3P1 overexpression reduced mice lung injury through negatively modulating miR‐21

**FIGURE 4:**
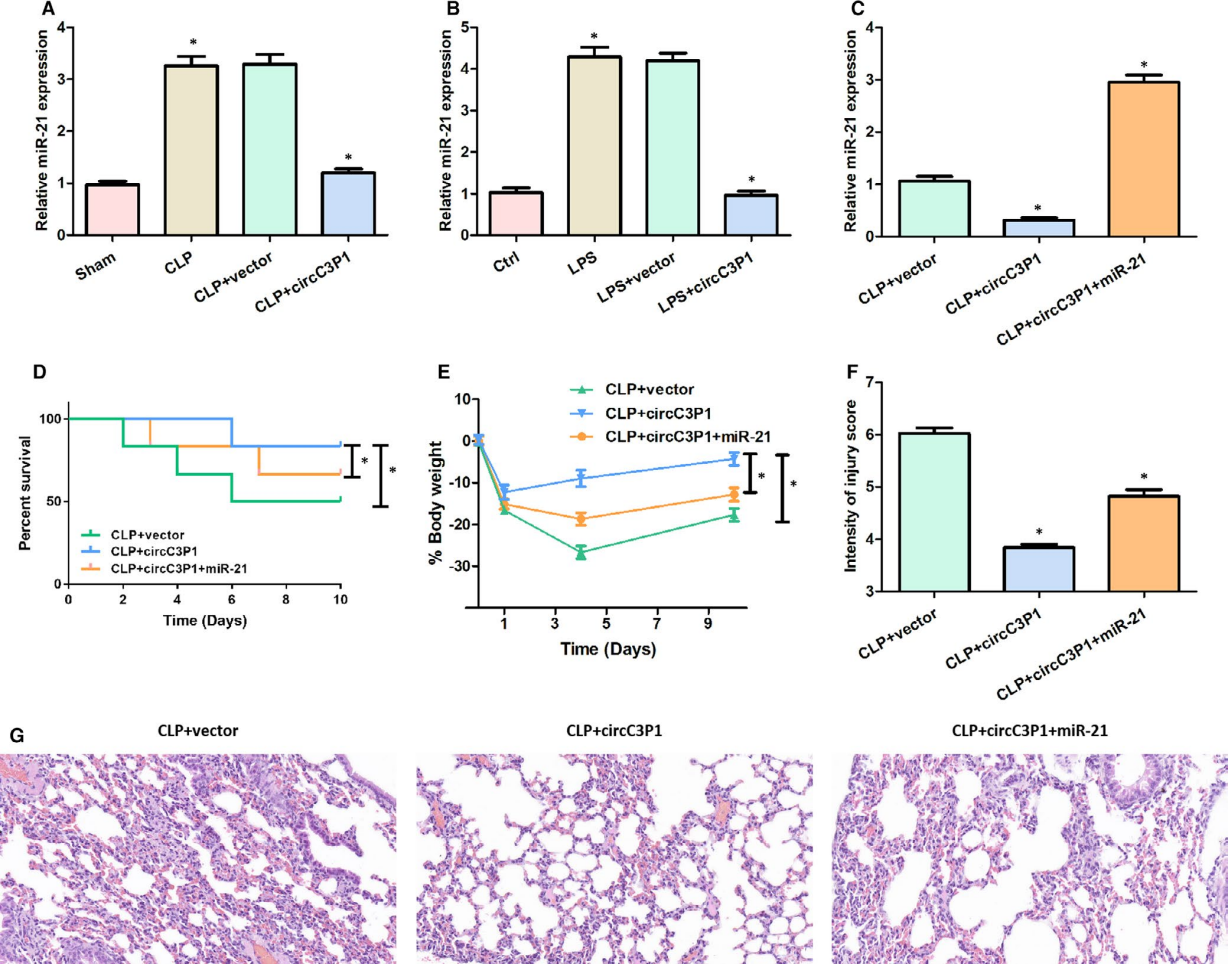
CircC3P1 overexpression reduced mice lung injury through negatively modulating miR‐21. A‐C, Relative miR‐21 expression in each group. D, Per cent survival in four groups. E, Body weight in three groups. F, Intensity of injury score in three groups. G, Representative histological images in three groups.*P < 0.05

As presented in Figure 4A,B, the relative miR‐21 expression was significantly higher in the CLP + vector group and the LPS + circC3P1 group than the Sham group and the control group, whereas the miR‐21 level was remarkably reduced after transfecting circC3P1. In Figure 4D,E, the per cent survival and body weight were evidently increased in the CLP + circC3P1 group, compared with the CLP + vector group and the CLP + circC3P1+miR‐21 group. In Figure 4C,F, the relative miR‐21 expression and injury score intensity were significantly decreased in the CLP + circC3P1 group than the CLP + vector group, while miR‐21 level and injury score intensity were evidently increased after transfecting miR‐21. Additionally, HE results indicated that the mice lung injury was alleviated in the CLP + circC3P1 group, while the lung injury was aggravated after transfection with miR‐21, as demonstrated in Figure 4G. These results showed that CircC3P1 overexpression reduced mice lung injury through negatively modulating miR‐21.

### 3.5 CircC3P1 overexpression reduced cell apoptosis and pro‐inflammatory cytokines levels via down‐regulating miR‐21

**FIGURE 5:**
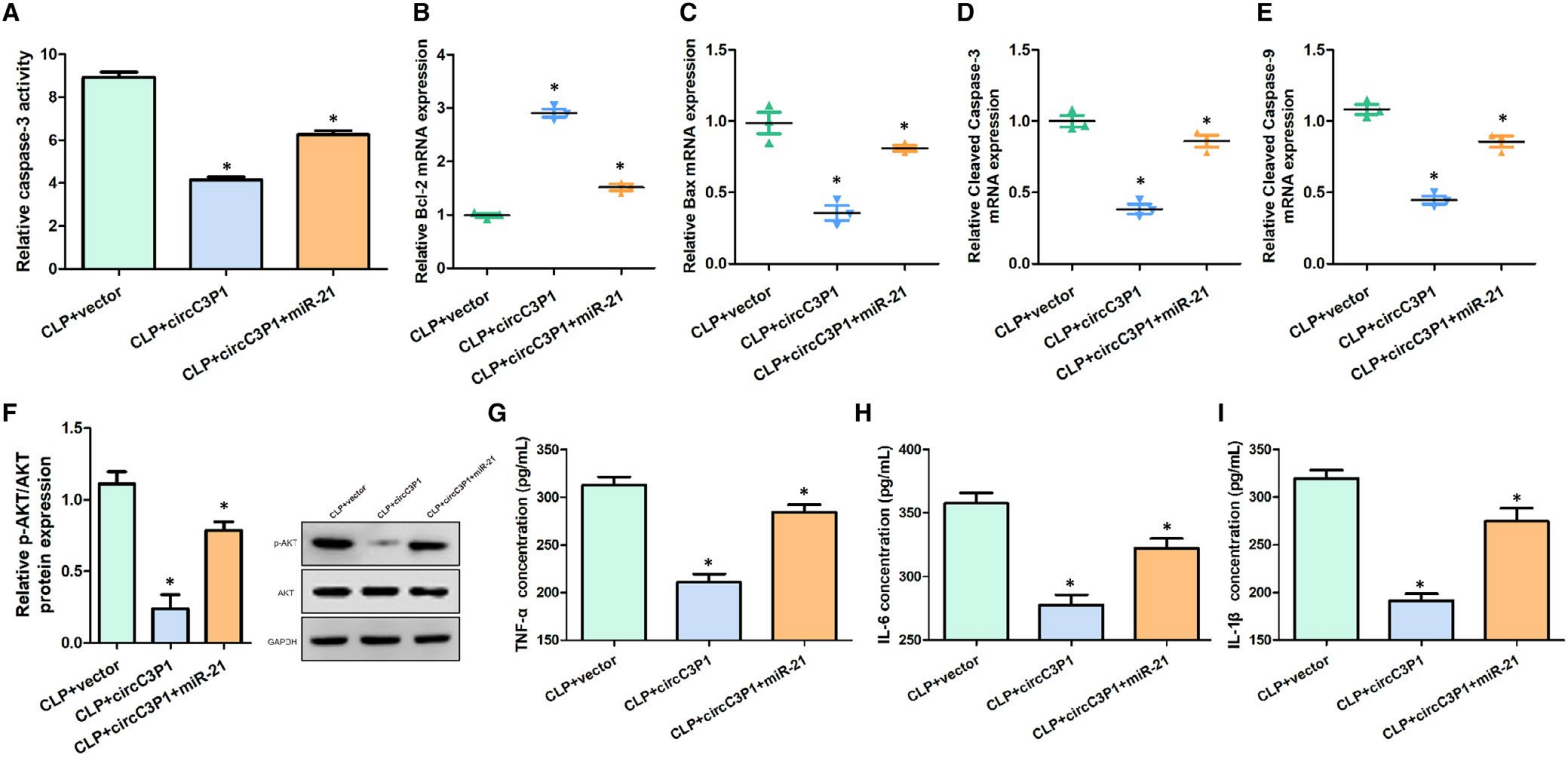
CircC3P1 overexpression reduced cell apoptosis and pro‐inflammatory cytokines levels via down‐regulating miR‐21. A, Relative caspase 3 activity in three groups. B, Relative Bcl‐2 mRNA expression in three groups. C, Relative Bax mRNA expression in three groups. D, Relative cleaved caspase 3 mRNA expression in three groups. E, Relative cleaved caspase 9 mRNA expression in three groups. F, The expression of p‐AKT and AKT protein in three groups. G‐I, The concentration of TNF‐α (G), IL‐6 (H) and IL‐1β (I) in three groups.*P < 0.05

Compared with the CLP + vector group and the LPS + vector group, the relative caspase 3 activity, relative Bax, cleaved caspase 3 and cleaved caspase 9 mRNA expression were significantly reduced, the relative p‐AKT/AKT protein expression and pro‐inflammatory cytokines levels (TNF‐α, IL‐6 and IL‐1β) were remarkably down‐regulated, and relative Bcl‐2 protein expression was markedly increased in the CLP + circC3P1 group and the LPS + circC3P1 group, while transfecting miR‐21 reversed this effect, as presented in Figure 5A‐I. Therefore, circC3P1 overexpression reduced cell apoptosis and pro‐inflammatory cytokines levels via down‐regulating miR‐21.

## 4 DISCUSSION

In the current study, we found that circC3P1 alleviated ALI induced by sepsis in vitro and in vivo. Besides, circC3P1 induction inhibited the expressions of pro‐inflammatory cytokines, such as TNF‐α, IL‐6 and IL‐1β, in the lung tissues of septic mice. Furthermore, circC3P1 overexpression decreased cell apoptosis, p‐AKT/AKT expression and pro‐inflammatory cytokines levels in MPVECs treated with LPS via down‐regulating miR‐21.

;lThe mouse CLP model is the most frequently used animal model of polymicrobial sepsis, due to the similar pathophysiology of human sepsis.^23^ CLP‐induced ALI mice model was successfully established in our study. The lung tissues indicated severe morphological changes, including thickening of alveoli, haemorrhage and alveolar collapse in the CLP group, compared with the sham group. In the sepsis‐induced ALI development, increasing pro‐inflammatory mediators and cell apoptosis play a significant role.^24^ Additionally, in ALI, pro‐inflammatory cytokines, including TNF‐α, IL‐6 and IL‐1β, are secreted for the cascade of inflammatory reactions.^25^ The suppression of cell apoptosis and inflammation may improve sepsis‐induced lung injury. LPS is a lipid endotoxin of Gram‐negative bacteria, which is commonly utilized to induce ALI in experiments.^26^ In a vitro model, MPVECs treated with LPS resulted in significant increase inflammatory cytokine levels and induction of cell apoptosis than those in the control group.

Our study indicated that relative circC3P1 expression was remarkably decreased in mice undergoing CLP operation than the sham‐operated mice. Also, circC3P1 dramatically attenuated pulmonary injury, decreased pro‐inflammatory cytokines (TNF‐a, IL‐6 and IL‐1β), cell apoptosis and p‐AKT/AKT levels induced by sepsis. This study also showed that circC3P1 might serve its protective role via anti‐apoptotic and anti‐inflammatory function in MPVECs treated with LPS. Presently, the data on the expression changes of circRNAs in ALI induced by LPS mice model are not enough. Li *et al* observed that five significantly increased (mmu_circRNA_44123, 42341, 44122) and decreased circRNAs (mmu_circRNA_25030 and 010498) in ALI model mice, compared with normal mice via cricRNA microarray analysis.^14^ To the best of our knowledge, the study presented in this report is the first investigation to explore the role of circC3P1 in ALI induced by sepsis. Such findings may offer important insights into the treatment of ALI.

In summary, circC3P1 attenuated pro‐inflammatory cytokine production and cell apoptosis in ALI induced by sepsis via down‐regulating miR‐21. This study indicates that circC3P1 is a promising therapeutic biomarker for sepsis‐induced ALI.

## CONFLICT OF INTEREST

The authors confirm that there are no conflicts of interest.

## AUTHOR CONTRIBUTION

**Wen Yang Jiang:** Conceptualization (equal); Writing‐original draft (equal). **Jie Ren:** Conceptualization (equal); Writing‐review & editing (equal). **Xing Hua Zhang:** Conceptualization (supporting); Writing‐original draft (supporting). **Zi Long Lu:** Investigation (equal); Project administration (equal). **Haojie Feng:** Investigation (supporting); Project administration (supporting); Writing‐review & editing (supporting). **Xiao Li Yao:** Investigation (equal); Project administration (equal). **Dong Hang Li:** Investigation (equal); Project administration (equal). **Rui Xiong:** Data curation (equal); Software (equal). **Tao Fan:** Data curation (equal); Software (equal). **Qing Geng:** Conceptualization (equal); Supervision (lead); Writing‐review & editing (equal).

## Supporting information

Table S1

## Data Availability

Data available on request from the authors.
