# Supplementary material for: CircC3P1 attenuated pro‐inflammatory cytokine production and cell apoptosis in acute lung injury induced by sepsis through modulating miR‐21
Source: J Cell Mol Med. 2020 Aug 26;24(19):11221–9. doi: 10.1111/jcmm.15685 (PMC7576301; doi:10.1111/jcmm.15685)
Supplement: Supplementary file 1 — Table S1 [file JCMM-24-11221-s001.docx]

**Table**

**Table S1. The primers utilized in this study**

| **Primers** | |
| --- | --- |
| circC3P1 (Forward ) | GGCCGGTACAATGAGACACA |
| circC3P1 (Reverse) | CGCATTCAGGTGGTACAGGT |
| p-AKT/AKT (Forward) | GTCAAGGCTGAGAACGGGAA |
| p-AKT/AKT (Reverse) | TCACGTTGGTCCACATCCTG |
| miR-21 (Forward) | UAGCUUAUCAGACUGAUGUUGA |
| miR-21 (Reverse) | AACAUCAGUCUGAUAAGCUAUU |
| Bax (Forward) | TGCAGAAGGATGATTGCTGAC |
| Bax (Reverse) | CACGCCATCCTCTCCAGAT |
| Bcl-2 (Forward) | AAGCTGTCCACAGGAGGGCTA |
| Bcl-2 (Reverse) | CACAGAGCTCATGTGTCCCAC |
| β-Actin (Forward) | CGAAACTACCTTCAACTCCATCATG |
| β-Actin (Reverse) | GCAATGATCTTGATCTTCATTGTG |
